# Removal of the Entire Tongue Successfully during Life

**Published:** 1866-08

**Authors:** 


### REMOVAL OF THE ENTIRE TONGUE SUCCESSFULLY DURING LIFE.

Mr. Nunnely exhibited to the Pathological Society a specimen of the entire tongue, which had been removed nineteen days before, from a man aged thirty-five years, by a submental opening.

He never had a bad system, and is now quite well.

The disease, which had existed sixteen or eighteen months, became worse two months before the operation, and from the pain and difficulty of speaking, the impossibility of mastication, and difficulty of deglutition, was fast wearing the patient out. He has already recovered strength and flesh; indeed, he says that he is as well as he ever was. He talks with great distinctness and swallows with facility.—*Med. Times and Gazette.*—*American Journal, July*, 1865.

